# Variation in the early-life interdigital skin microbiota of lambs in a commercial sheep flock

**DOI:** 10.3389/fvets.2026.1923848

**Published:** 2026-09-30

**Authors:** Katharine Lewis, Marisa Draycott, Laura Green, Rachel Clifton

**Affiliations:** 1 School of Veterinary Medicine and Science, University of Nottingham, Nottingham, United Kingdom; 2 School of Biosciences, University of Birmingham, Birmingham, United Kingdom

**Keywords:** early life, footrot, interdigital skin, lamb, microbiome

## Abstract

**Introduction:**

The factors shaping early-life microbial colonisation of the ovine interdigital skin, and their potential relevance to foot health and footrot susceptibility, remain poorly understood. Here, we investigated variation in the interdigital skin microbiota among lambs in different age-location groups.

**Methods:**

Interdigital skin swabs were collected from all four feet of 25 lambs aged 0–15 days on a commercial farm. Bacterial community composition was characterised using 16S rRNA gene amplicon sequencing, and quantitative PCR was used to estimate total bacterial load and detect *Dichelobacter nodosus*.

**Results:**

Community composition differed markedly among lamb age-location groups. Microbial communities were more tightly clustered in newborn lambs than in older lambs and total bacterial load was higher in lambs ≥ 1 day old. A consistent early-life core microbiome, dominated by *Staphylococcaceae* and *Corynebacteriaceae*, was detected across all groups, although dominant amplicon sequence variants (ASVs) differed among age-location groups. *D. nodosus* was not detected in any lambs; however, ASVs assigned to the genus *Porphyromonas* were detected in the interdigital skin microbiota at birth.

**Discussion:**

These findings demonstrate substantial variation in the early-life interdigital skin microbiota among lamb age-location groups, providing a baseline for future studies investigating microbial community development and ovine footrot.

## 1 Introduction

Early life influences can have long-lasting effects on health and disease risk, a concept formalised in the Developmental Origins of Health and Disease Hypothesis (1). One such influence is the early life microbiome and the process by which it establishes (2). The early life infant microbiome is in many cases established via maternal seeding events (3–5), which can include environmental transfer of bacteria that are associated with disease (3, 6). However, the extent of persistence of these maternally seeded microbiomes into later life varies, with key windows of infant development often coinciding with changes in their microbiome. These developmental windows can be associated with both internal events, such as development of the immune system, illustrated by the induction of key immune components in piglets at approximately two weeks of age (7) and external events, such as diet changes or antibiotic administration in human infants (5, 8). Together, these findings suggest that early-life microbial assembly, including that of livestock species, is shaped by both maternal and environmental factors and might influence susceptibility to infectious diseases later in life.

In sheep, the interdigital skin microbiota (IDSM) plays a key role in the pathogenesis of ovine footrot, a contagious dermatitis with substantial welfare and economic implications. Footrot is present in >99% of flocks in England (9) and accounts for around 70% of lameness cases (10, 11). Footrot is a polymicrobial disease in which *Dichelobacter nodosus* is recognised as the essential causative agent, often acting in synergy with *Fusobacterium necrophorum* (12, 13). However, increasing evidence indicates that clinical manifestations of footrot are not solely driven by these pathogens, but arise from more complex interactions within the wider bacterial community on the foot. Several studies have reported altered microbial diversity in footrot-affected feet, alongside an increased prevalence of *Porphyromonas* spp., *Treponema* spp., and *Mycoplasma fermentans* (14–16). Supporting the importance of this broader community, the composition of the IDSM in adult ewes, and particularly the presence of *Porphyromonas* spp., has been shown to be predictive of subsequent footrot events (17).

Despite this understanding in adult sheep, little is known about how the IDSM is established in early life or how it varies between individuals. Some cases of footrot in young lambs have been associated with cases in their mother, likely because close and prolonged spatial contact increases the probability of environmental transfer of disease-associated bacteria from ewes to their lambs (18). Farmers also report that lambs with lame mothers are likely to become lame (19). Based on principles of early-life microbial assembly and evidence for maternal involvement in transmission, it is plausible that the initial establishment of the lamb IDSM influences later susceptibility to footrot.

Currently, it is not known whether bacterial taxa in the IDSM that are associated with increased footrot susceptibility in ewes (17) are present in early-life. The only available evidence indicates that *D. nodosus* is absent at birth but can be detected within the first 24 h of life in flocks with ewes lame with footrot (6). Beyond this, early community assembly and the presence of commensal or potentially pathogenic taxa in young lambs remain undocumented. Farmers report that lambs are more likely to present with early-stage footrot (interdigital dermatitis), while severe lesions are less common than in ewes (10, 20). This suggests potential differences in pathogenesis between age groups, possibly linked to repeated exposure and chronic inflammation.

Understanding early IDSM assembly in lambs is therefore critical for identifying potential windows for intervention (e.g., management or husbandry approaches) and for determining whether early microbial exposure contributes to lifetime footrot risk. The aim of this study was to examine the interdigital skin microbiota of lambs aged 0–15 days, to investigate variation in community composition among lamb age-location groups, and to determine the occurrence of key footrot-associated bacterial taxa within this early-life microbial community.

## 2 Materials and methods

### 2.1 Ethical approval and informed consent

All procedures were approved by the University of Birmingham Animal Welfare and Ethical Review Body on 11 March 2022 (Reference number: Clifton_UoB AWERB Approval_Investigating the role of the early-life interdigital skin (IDS) microbiota in susceptibility of sheep to footrot_11.03.2022). The participating farmer provided written informed consent for the involvement of ewes and lambs, and all animals remained under standard farm management throughout the study.

### 2.2 Animals and experimental design

This was a cross-sectional observational study conducted on a single commercial sheep farm. The flock had 554 breeding ewes and low levels of lameness, with 50 ewes lame over one year at the time of the study. Twenty-five healthy lambs aged 0–15 days were opportunistically sampled during a single visit to the farm in March 2022. Metadata, including lamb sex, ewe identity, and housing location at the time of sampling were recorded from farm records (Table 1; Supplementary Table 1).

**Table 1:** Descriptive summary of lambs sampled during the single farm visit.

Age group	Location	Number of lambs	Number of ewes	Sex		Twin-pair in study*	
				Female	Male	No	Yes
0 days postpartum	NA	5	3	0	5	1	4
1–4 days	Individual pen	10	9	5	5	8	2
8–15 days	Individual pen	4	2	3	1	0	4
	Pasture	6	6	4	2	6	0
Total		25	20	12	13	15	10

Four lambs from two ewes had spent time at pasture but were returned to individual pens due to suspected mastitis in their ewes.

*Ten lambs were from twin pairs where both twins were sampled; for others, twin status was not confirmed but only one lamb per ewe was sampled.

Prior to lambing, ewes were housed indoors on straw in group pens. Following parturition, ewe-lamb(s) groups were transferred into individual pens that were cleaned and disinfected between occupants. These family units remained indoors for up to five days depending on their health status, weather conditions, and pen availability, before being turned out to pasture. Animals at pasture were monitored daily, and any ewes showing signs of ill-health, such as suspected mastitis, were returned to individual pens with their lambs.

### 2.3 Sample collection

The interdigital skin of all four feet of each lamb was sampled using sterile nylon flocked swabs (COPAN Italia S.p. A., Brescia, Italy) following the method described by Clifton et al. (21). Each swab was passed five times across the interdigital skin. Lambs sampled at birth (newborn, 0 day-old) were caught before contacting the ground, and foetal membranes were removed when necessary. Lambs aged 1–4 days were sampled in individual pens, whereas lambs aged 8–15 days were sampled either in individual pens or at pasture depending on where they were located at the time of sampling. Swabs were immediately placed into 1.8 mL cryotubes (Corning Incorporated, Corning, NY, USA) containing 1 mL RNAlater® (Thermo Fisher Scientific, Waltham, MA, USA) and stored at −80 °C until analysis.

### 2.4 Laboratory methods

#### 2.4.1 DNA extraction

DNA was extracted using the ZymoBIOMICS DNA/RNA Miniprep Kit (Zymo Research, Irvine, CA, USA), further details of extraction steps are in Supplementary material S1. The mechanical lysis step consisted of a 2.5-min bead-beating period, a 1-min pause, and a further 2-min bead-beating period. Samples and all controls (detailed in Section 2.3.4) were processed in parallel. Extracted DNA was stored at −20 °C prior to sequencing and qPCR analysis.

#### 2.4.2 Quantification of total bacterial load and *Dichelobacter nodosus*

Total bacterial load (TBL) was quantified using SYBR Green quantitative PCR targeting the 16S rRNA gene with primers SD-17F and SD-21R (22), full details of the reaction protocols are in Supplementary material S2. Each reaction was performed in duplicate, and gene copy numbers were calculated per swab. The presence and quantity of *Dichelobacter nodosus* was assessed using a TaqMan qPCR targeting the *rpoD* gene (23), also run in duplicate. Samples were classified as positive only when both technical replicates amplified. Full sequences of all primers used are in Supplementary Table 2.

#### 2.4.3 16S rRNA gene amplification and sequencing

Library preparation steps for the 16S rRNA gene sequencing are in Supplementary material S3. The V3–V4 region of the bacterial 16S rRNA gene was amplified using the SD-17F and SD-21R primers (22). PCR products were submitted to Deep Seq, University of Nottingham, for library preparation using the Illumina 16S Metagenomic Sequencing Library Preparation protocol. Sequencing was performed on an Illumina MiSeq platform using v3 600-cycle chemistry to generate paired-end 300 bp reads.

#### 2.4.4 Laboratory control methods

To monitor potential contamination and verify performance throughout the workflow, sampling, extraction, and PCR controls were included alongside all samples. A blank swab placed in RNAlater® (Thermo Fisher Scientific) served as a sampling negative control. A DNA Shield tube processed without a swab was included as an extraction negative control in every extraction batch. A ZymoBIOMICS Microbial Community Standard (Zymo Research) was used as a positive extraction control. PCR plates included a ZymoBIOMICS microbial DNA standard (Zymo Research) as a PCR positive control and nuclease-free water as a PCR negative control.

### 2.5 Bioinformatic methods

All bioinformatic processing was conducted using R version 4.5.2 and R Studio version 2025.09.2 + 418 (24). Raw sequence data were processed using the DADA2 pipeline (25) to generate amplicon sequence variants (ASVs). Quality control steps used were removal of primers from sequences and truncation of reverse reads by 50 bp. All reads were truncated at the first instance of a quality score ≤ 2 and sequences were further quality filtered using a maximum expected error rate of 2 for forward reads and 5 for reverse reads. Denoising was performed to learn error rates for each read and infer ASVs, with pairs of denoised forward and reverse reads merged (minimum overlap: 12 bp, maximum mismatch rate: 0). Chimeras were identified by the consensus across samples method and removed.

Taxonomic assignment was performed using the SILVA 138 SSU Ref NR99 database. ASVs detected in any negative control were removed and positive controls were evaluated for whether they contained the expected taxonomic composition. Four samples with < 1,000 reads were excluded from downstream analyses and rarefaction was performed to confirm whether adequate sequencing depth had been achieved for the remaining 96 samples (Supplementary Figure S1).

To identify key taxa, ASVs assigned to the genera *Porphyromonas* and *Treponema* were aligned with curated reference sequences from GenBank using MAFFT v7.526 (26). Phylogenetic trees were generated using FastTree v2.1.10 (27) and visualised in R using the *ggtree* package (28). MAFFT and FastTree were both run on the Galaxy server (usegalaxy.org) (29) with default parameters. As only a small number of ASVs were assigned to the genera *Fusobacterium* and *Mycoplasma*, these were compared to reference sequences using the megablast algorithm (30) to determine their similarity to *Fusobacterium necrophorum* and *Mycoplasma fermentans*, respectively.

### 2.6 Statistical methods

Three linear mixed effects models using *lme4* (31) were used to model (i) TBL_log10_ (Model 1) and (ii) within-foot community α-diversity. α-diversity was summarised by the number of ASVs (Model 2) and the Shannon Index (Model 3) using *Phyloseq* (32). The observed number of ASVs was square root transformed, and the Shannon Index was reflected square root transformed using a value of 10, to give a linear outcome variable. On the reflected Shannon Index scale, higher values correspond to communities with lower richness and evenness. One outlier was excluded here (>1,000 ASVs, range of all others was 25–418) (Figure 1a). Fixed effects predictors included in the model were lamb sex, age, whether their twin pair was included in the study and age-location group. Lamb identity was included as a random effect to account for sampling four feet from each lamb. Model fit was assessed by visualisation of residuals for linearity, normality and homoscedasticity. A joint test of equality of slopes across quantiles (τ = 0.1, τ = 0.5, τ = 0.9) was used to test whether predictor effects were stable across the distribution of α-diversity metrics using a linear model of the fixed effects predictors using *quantreg* (33). To account for clustering at lamb level, quantile regression models were fitted with robust standard errors using a cluster bootstrap with 500 replicates, with lamb specified as the clustering unit.

**Figure 1:**
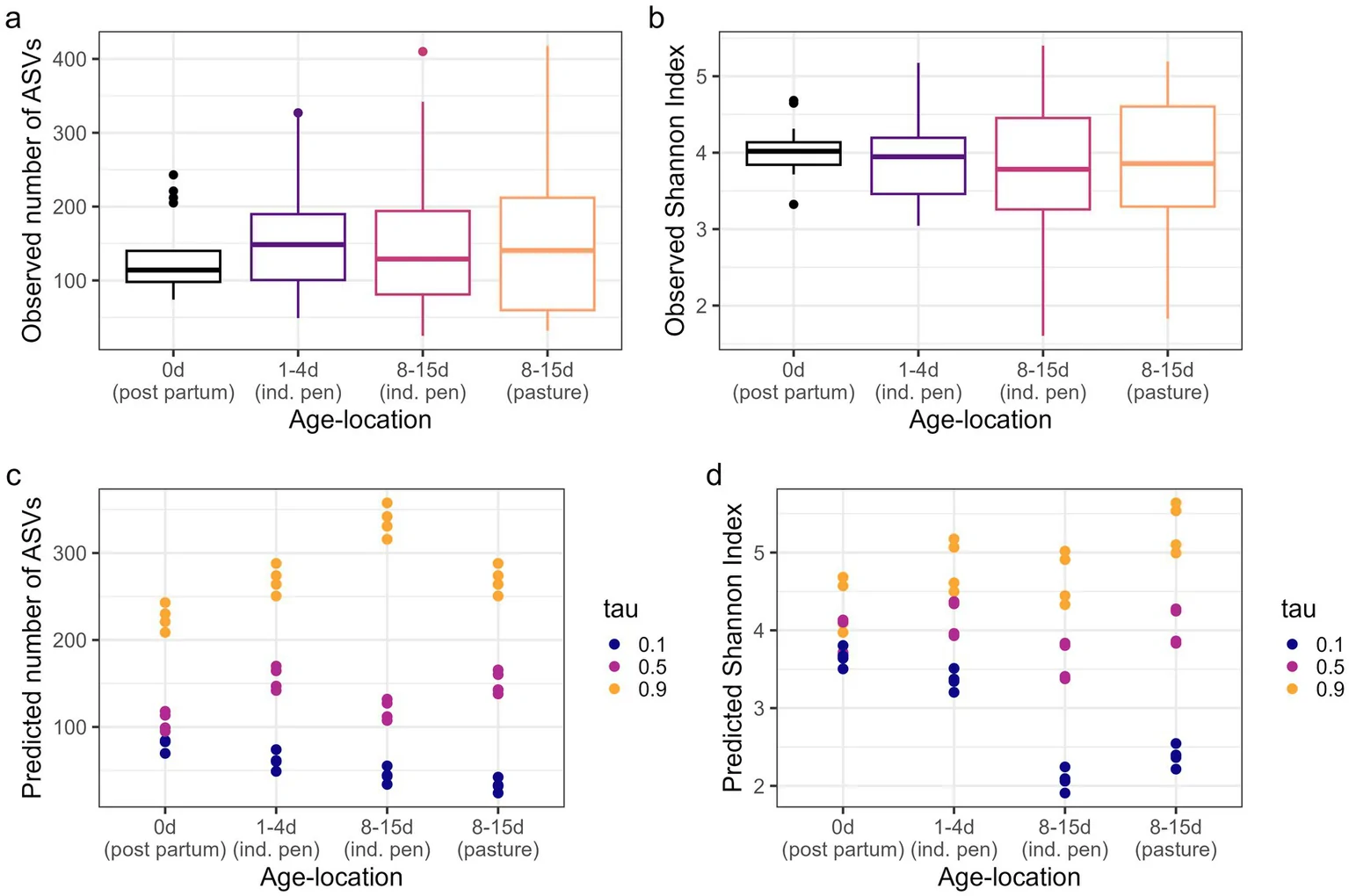
Observed values of α-diversity metrics within age-location groups and predicted values from linear models at different quantiles of the distribution of α-diversity metrics (tau = 0.1, 0.5, 0.9) for **(a,c)** number of ASVs and **(b,d)** Shannon Index indicating heterogeneity between age-location group, sex and twin-status on α-diversity of the lamb foot microbiome. A joint test of equality of slopes revealed significant differences between slopes at tau = 0.1 and 0.9 for both number of ASVs (*p* = 0.002) and Shannon index (*p* = 0.001).

To summarise between-foot community diversity (β-diversity), two dissimilarities matrix indices (Bray-Curtis, which measures compositional dissimilarity based on species abundance and Jaccard, which uses species presence–absence) were calculated from ASV relative abundances in *Phyloseq* (32). Both matrices range from 0 (indicating identical communities) – 1 (indicating no common species). Patterns of community structure based on the two dissimilarity matrices were visualised using non-metric multidimensional scaling (NMDS).

Differences in community structure between lambs were assessed using permutational analysis of variance (PERMANOVA) with 999 permutations within *vegan* (34). To account for non-independence of observations within lambs, the four foot samples from each lamb were aggregated into a single lamb-level community profile prior to calculating between-sample distances for PERMANOVA. Sample raw abundances were aggregated by lamb using the *merge_samples* function in *Phyloseq* which sums abundances for each ASV. Relative abundance of each ASV was then calculated per lamb and Jaccard distance calculated from relative abundances as described above. The PERMANOVA accounted for age-location group, sex, and whether an individual’s twin was also included in the study. To determine whether any observed differences were attributable to variation in dispersion rather than shifts in group centroids, beta-dispersion was evaluated using a test of multivariate homogeneity (PERMDISP) using *vegan* (34). Beta-dispersion was evaluated with samples aggregated to lamb level.

### 2.7 Characterisation of bacterial family abundance and known footrot associated taxa

Heatmaps were used to visualise the prevalence of bacterial families across age-location groups. To improve visualisation of differences among groups and reduce the influence of taxa detected only at very low relative abundance, bacterial families were considered present only when they exceeded 1% relative abundance within a sample. This threshold was applied for prevalence visualisation only and did not affect downstream statistical analyses. Relative abundance of dominant bacterial genera was compared between age-location groups using stacked bar plots. For taxa associated with footrot pathogenesis, including *D. nodosus*, *F. necrophorum*, *Porphyromonas*, *Treponema*, and *Mycoplasma fermentans*, prevalence, abundance, and the number of ASVs assigned to each taxon were summarised.

## 3 Results

### 3.1 Total bacterial load, within foot community diversity, and associations with age-location group

TBL was detected in 97 of 100 samples and ranged from 3.81 × 10^5^ to 7.46 × 10^9^ gene copies per swab (Table 2). TBL_log10_ (Model 1) was lowest in 0 day old lambs and significantly higher in lambs of 1–4 days old (β = 0.92, 95% CI = 0.27–1.57) in individual pens, 8–15 days old at pasture (β = 1.29, 95% CI = 0.49–2.08) and those that had returned to pens, i.e., those where the mothers were unwell (β = 0.82, 95% CI = 0.17–1.57) when compared with TBL_log10_ of 0-day old lambs.

**Table 2:** Summary of number of lambs, number of quantifiable samples, and total bacterial load from interdigital skin swabs for four age-location groups of lambs.

Age group	Location	N_lambs_	N_quantifiable samples_	Median TBL (SD) IQR
0 days (post partum)	NA	5	18	6.24 × 10^6^ (4.38 × 10^7^) 1.72 × 10^6^–3.27 × 10^7^
1–4 days (early neonatal)	Individual pen	10	40	2.73 × 10^7^ (1.44 × 10^8^) 4.38 × 10^6^–6.13 × 10^7^
8–15 days (early neonatal)	Individual pen*	4	16	6.35 × 10^7^ (2.43 × 10^8^) 1.15 × 10^7^–2.55 × 10^8^
	Pasture	6	23	2.91 × 10^7^ (1.79 × 10^8^) 5.83 × 10^6^–1.77 × 10^8^
Total		25	97	2.50 × 10^7^ (9.01 × 10^8^) (4.18 × 10^6^–9.51 × 10^7^)

N = number, TBL = total bacterial load, SD = standard deviation, IQR = interquartile range, NA = not applicable; 0-day lambs were sampled before touching the ground.

*Four lambs from two ewes had spent time at pasture but were returned to individual pens due to suspected mastitis in their ewes.

TBL_log10_ was also higher in lambs where it was known their twin was included in the study (β = 0.63, 95% CI = 0.05–1.20). No variation in TBL_log10_ was attributed to lamb identity after accounting for age-location, sex and whether their twin was in the study (Model 1: 0.0%, Table 3).

**Table 3:** Linear mixed effects models for total bacterial load and the two α-diversity indices (number of ASVs and Shannon Index) and their association with age and location, sex and if a lamb’s twin was known to be in the study.

Model predictors and performance	Model 1: TBL_log10_ (N_samples_ = 97)β (LCI-UCI)	Model 2: N_ASVsq_ (N_samples_ = 95)β (LCI-UCI)	Model 3: Shannon Index_reflected-sq_ (N_samples_ = 95) β (LCI-UCI)
Intercept	6.25 (5.45–7.06)	10.62 (5.84–15.4)	2.53 (2.31–2.75)
Fixed effects			
Age-location			
0 days – post partum	Reference	Reference	Reference
1–4 days – individual pen	0.92 (0.27–1.57)	1.29 (−2.57–5.16)	−0.05 (−0.23–0.13)
8–15 days – individual pen	0.82 (0.17–1.47)	−0.12 (−3.97–3.73)	0.08 (−0.1–0.26)
8–15 days – pasture	1.29 (0.49–2.08)	0.94 (−3.78–5.66)	−0.06 (−0.28–0.16)
Sex			
Female	Reference	Reference	Reference
Male	0.12 (−0.29–0.54)	−0.28 (−2.76–2.19)	−0.00 (−0.12–0.12)
Twin pair included in study			
No	Reference	Reference	Reference
Yes	0.63 (0.05–1.20)	1.23 (−2.19–4.65)	−0.11 (−0.27–0.05)
Random effects	Variance	Variance	Variance
Lamb	0.00	3.50	0.01
Residual	0.72	8.96	0.01
Model performance			
Conditional, marginal R^2^	0.14, NA	0.30, 0.02	0.43, 0.06
ICC	NA	0.28	0.39

TBL = total bacterial load, N_ASV_ = number of ASV, sq = square root scale, N_samples_ = number of samples, β = model coefficient, LCI = lower confidence interval, UCI = upper confidence interval, ICC = intra-class correlation coefficient, Reference = model reference category.

NA for model performance measures indicates where a metric could not be calculated, as the random effect estimate was 0.

The median number of ASVs in the 96 samples was 133.5 (standard deviation (SD) = 128.38) and ranged from 25–1,116, while median Shannon index was 3.94 (SD = 0.74) and ranged from 1.60–5.40. The results from the linear mixed effects models of α-diversity, with foot results adjusted for within lamb correlation, were that richness and evenness of the foot bacterial communities did not differ in the mean number of ASVs (square root scale) (Model 2) or Shannon Index (reflected square root scale) (Model 3) between age-location groups (Table 3). Quantile-based regression indicated that the effects of age-location group, sex, and twin status on α-diversity were not consistent across all feet but were more pronounced at the lower and upper ends of the α-diversity distributions for both ASV number (Model 2: ͳ = 0.1&0.9, *p* = 0.002) and Shannon Index (Model 3: ͳ = 0. 1& 0.9, *p* = 0.001). In particular, at the upper end of the α-diversity distribution (Figures 1a,b), lambs aged 8–15 days, whether housed or at pasture, exhibited higher predicted numbers of ASVs than 0-day old lambs (Figure 1c). Together, these findings indicate that age and environmental exposure were associated with differing diversity bacterial communities at the ends of the α-diversity distribution rather than having uniform effects across all feet, as illustrated in Figures 1c,d. The random effect term for lamb identity was 3.50 (Model 2) and 0.01 (Model 3) which corresponded to explaining around 1/3rd of the remaining variance in α-diversity metrics once age-location group, sex and if their twin was in the study had been accounted for (Model 2: ICC = 28%, Model 3: ICC = 39%), illustrating considerable variation in the within foot community between individual lambs (Table 3). Lamb identity explained proportionally more of the variance than age-location group, sex and whether the twin pair was included in the study, the conditional R^2^ was higher than the marginal R^2^ in both models (Model 2: conditional R^2^ = 0.30, marginal R^2^ = 0.02, Model 3: conditional R^2^ = 0.43, marginal R^2^ = 0.06). These findings indicate that residual variation in α-diversity may arise from unmeasured host-level factors.

### 3.2 Between-community diversity, and associations with age-location group

NMDS ordination of β-diversity metrics revealed distinct clustering of IDSM bacterial communities from 0 day old and 1–4 day old lambs housed indoors compared with lambs 8–15 days old at pasture (Figure 2a). Similar clustering patterns were observed using both Bray-Curtis and Jaccard dissimilarity metrics, indicating that the observed differences among age-location groups were robust to the choice of β-diversity metric and reflected variation in both taxon composition and relative abundance.

**Figure 2:**
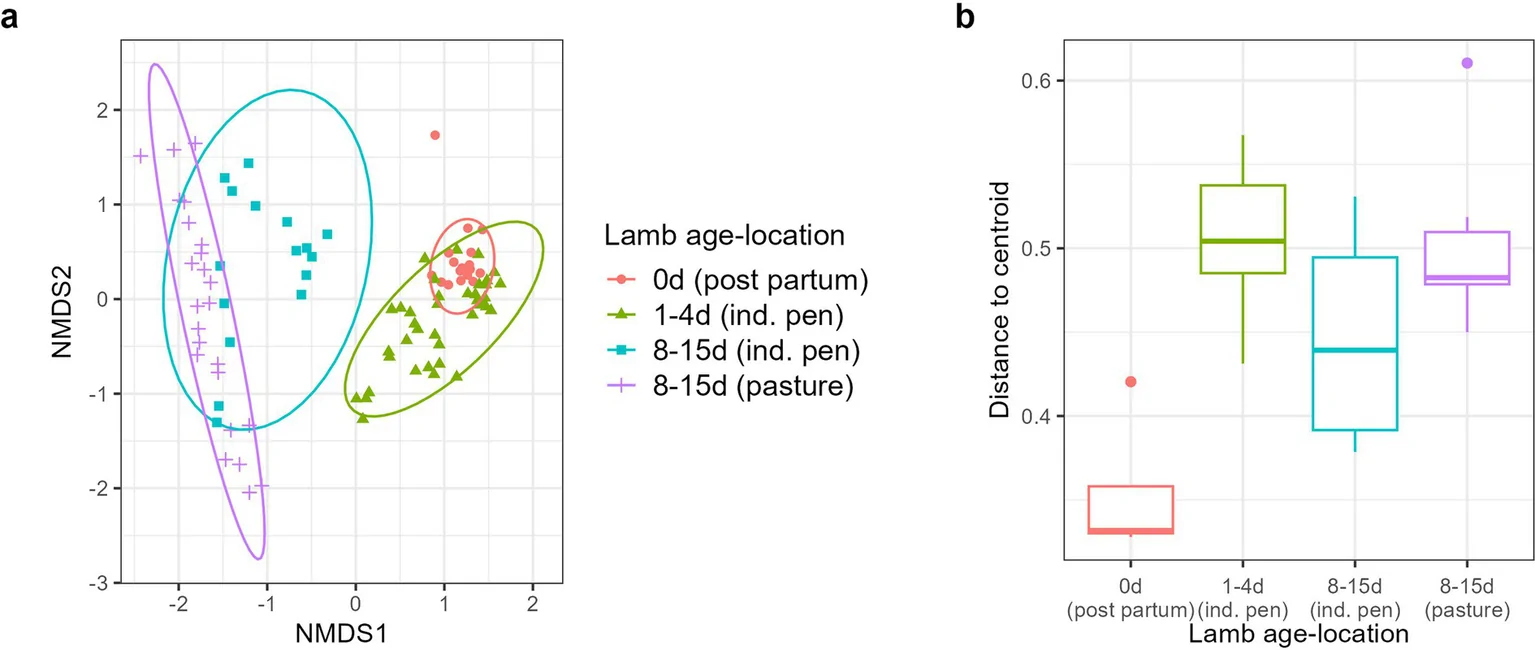
Composition of the interdigital skin microbiota of lambs by age-location group. **(a)** Non-metric multidimensional scaling (NMDS) ordination based on Jaccard dissimilarity of interdigital skin microbial communities. Data are visualised at the foot level; therefore each point represents an individual foot sample. Colour and shape indicate lamb age-location group. To account for non-independence among feet from the same lamb, statistical analyses were performed at the lamb level. PERMANOVA showed a significant effect of age-location group on community composition (pseudo-*F* = 4.296, R^2^ = 0.373, *p* = 0.001). **(b)** Distance of observations to their group centroid based on Jaccard dissimilarity, calculated at the lamb level, showing variation in within-group dispersion among age-location groups. PERMDISP indicated significant differences in dispersion between groups (*F* = 10.737, *p* = 0.001). Total number of samples (lambs) per group: 0d = 17 (5), 1–4d = 39 (10), 8–15 ind. pen = 16 (4), 8–15d pasture = 24 (6). Ind. pen = individual pen, d = days old.

Analysis of multivariate dispersion (PERMDISP) indicated significant differences in within-group dispersion among age-location groups (*F* = 10.737, *p* = 0.001). Specifically, 0 day old lambs had significantly lower within-group dispersion of IDSM bacterial communities than older lambs (differences in the mean distance of samples to their group centroid = −0.15 compared with 8–15 days old lambs at pasture and 1–4 day old lambs in an individual pen, −0.10 for 8–15 day old lambs that had returned to a pen, Figure 2b), indicating that bacterial communities from newborn lambs were more homogeneous than those from older lambs.

Despite these differences in dispersion, PERMANOVA indicated a significant association between age-location group and lamb microbial community composition based on Jaccard dissimilarity (pseudo-*F* = 4.296, R^2^ = 0.373, *p* = 0.001). Lamb sex, and whether a lamb’s twin was in the study were not significantly associated with dissimilarity in presence-absence of microbial species between lambs (pseudo-*F* = 1.042, *p* = 0.34 and pseudo-*F* = 1.599, *p* = 0.06 respectively). Together, age-location group, lamb sex and twin status explained 45% of variation in the Jaccard dissimilarity between lambs. Of the explained variation, 37.3% was attributed to age-location, 3.0% to lamb sex and 4.6% to twin status, suggesting age-location was an important influence on community composition at the lamb level. However, given the significant differences in within-group dispersion between age-location groups, the PERMANOVA findings should be interpreted as reflecting differences in both community composition and within-group dispersion.

### 3.3 Prevalence-based visualisation of the core microbial community

Core bacterial families were present across all age-location groups, these were predominantely from the families *Staphylococcaceae* and *Corynebacteriaceae* (Figure 3), indicating that these taxa form a stable early-life core of the IDSM. However, the prevalence of other bacterial families differed markedly with age-location group. Newborn lambs (0 day old) showed high prevalence of several families including *Dietziaceae*, *Bacillaceae*, and *Halomonadaceae*. In contrast, lambs aged 8–15 days sampled at pasture showed higher prevalence of gut-associated families such as *Oscillospiraceae*, *Rikenellaceae*, and *Prevotellaceae*. Lambs sampled indoors at 1–4 or 8–15 days showed intermediate profiles between these two extremes.

**Figure 3:**
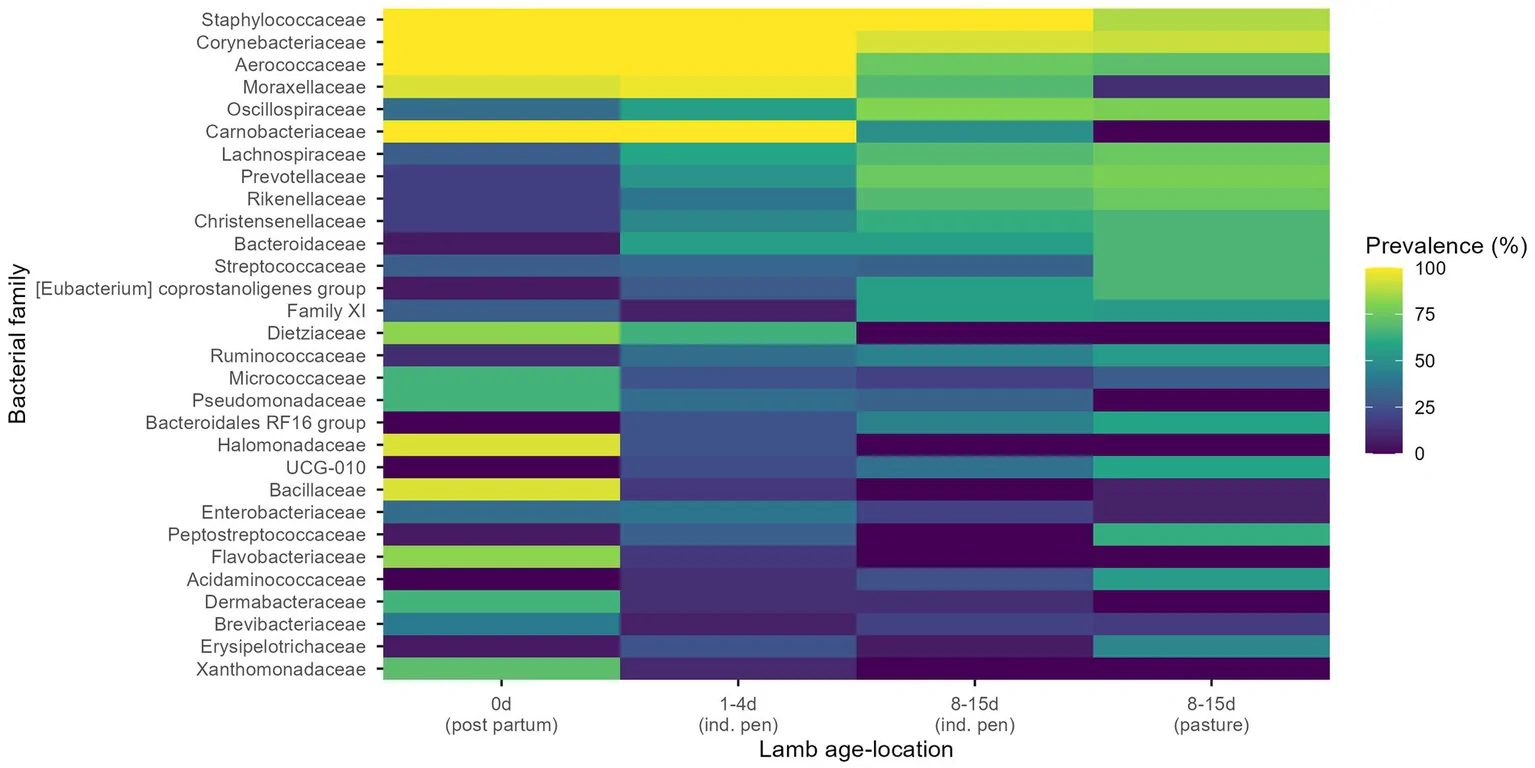
Heatmap showing the prevalence of the top 30 bacterial families in the lamb interdigital skin microbiota by lamb age-location. Prevalence was calculated as the number of samples where a bacterial family was present, as a percentage of the total number of samples from that group of lambs. To aid visualisation a > 1% relative-abundance threshold was used to define presence. Total number of samples per group: 0d = 17, 1–4d = 39, 8–15 ind. Pen = 16, 8–15d pasture = 24. Ind. pen = individual pen, d = days old.

### 3.4 Taxonomic composition of key bacterial families at the ASV level

Although *Staphylococcaceae* and *Corynebacteriaceae* were present in both newborn (0-day) and neonatal (1–4 days and 8–15 days) lambs, the dominant ASVs within these families differed by age-location group (Figure 4). In *Staphylococcaceae*, ASVs assigned to *Jeotgalicoccus* and *Staphylococcus* dominated in newborn and 1–4-day old lambs, whereas *Macrococcus* ASVs dominated in 8–15-day old lambs. In *Corynebacteriaceae*, all ASVs belonged to the *Corynebacterium* genus however ASV35 was detected exclusively in 8–15-day old lambs, while ASV2, ASV4, and ASV6 predominated in the younger lambs. These findings indicate fine-scale differences in ASV composition within major families across age-location groups.

**Figure 4:**
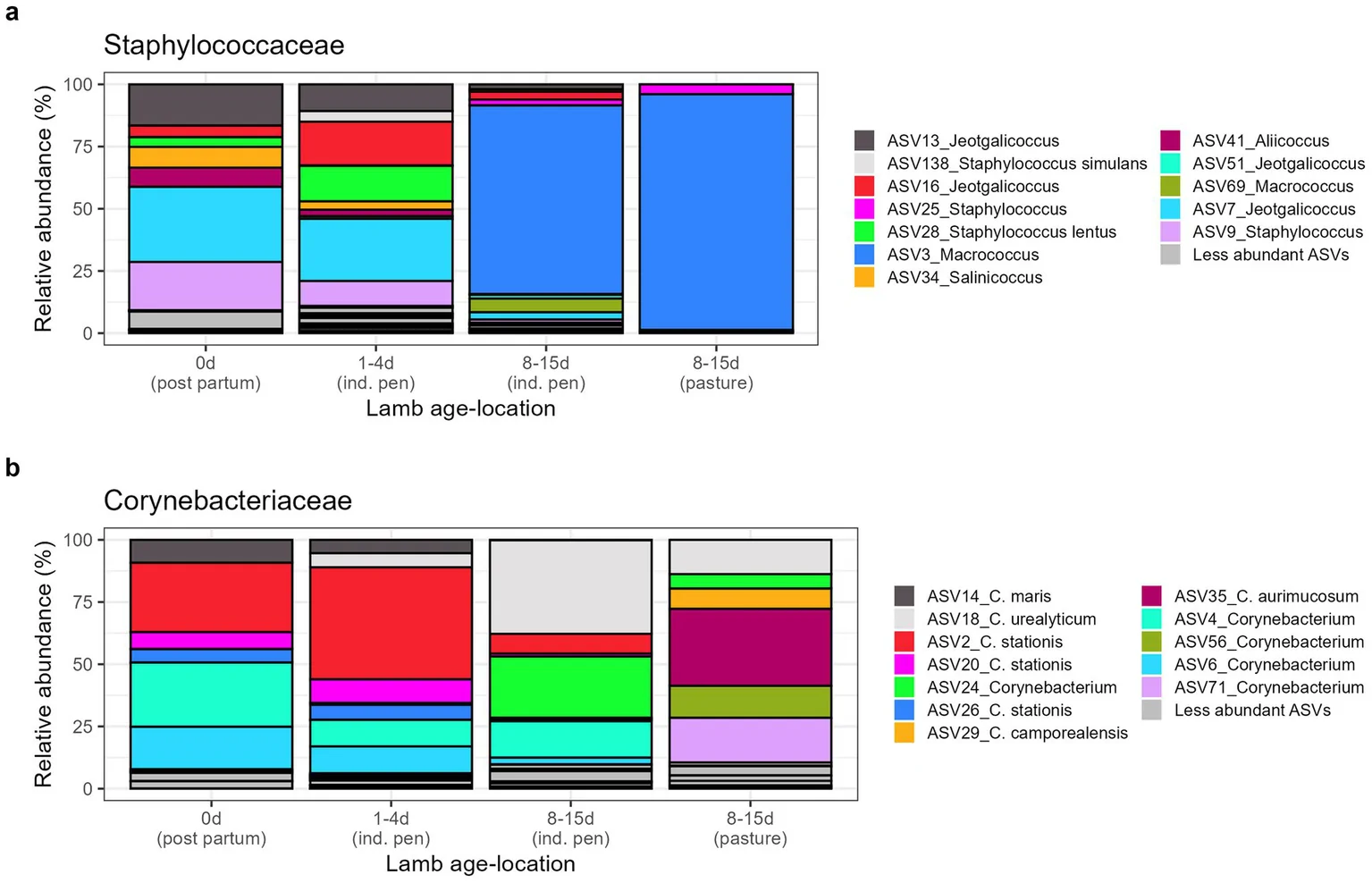
Presence and abundance of ASVs belonging to the **(a)** *Staphylococacceae* and **(b)** *Corynebacteriaceae* families in the interdigital skin microbiota of lambs. Abundance profiles are summarised by age-location of lambs and classification shown for ASVs is the highest resolution obtained from the SILVA database.

### 3.5 Presence, prevalence and abundance of bacteria previously associated with footrot pathogenesis

Nineteen ASVs were assigned to the genus *Porphyromonas*. Phylogenetic analysis of 16S rRNA sequences placed these ASVs close to *P. levii*, *P. somerae*, *P. asaccharolytica*, and *P. uenonis* (Figure 5a). Several *Porphyromonas* ASVs were detected in 0 day old lambs, consistent with immediate early-life acquisition, as well as being detected in 8–15 day old lambs (Figure 5b; Supplementary Figure S2)*. D. nodosus* was not detected in any sample by either qPCR or 16S rRNA gene sequencing. Two *Fusobacterium* ASVs showed >99% sequence identity to reference sequences of *Fusobacterium necrophorum*; these were detected at low prevalence in three samples from two lambs housed indoors.

**Figure 5:**
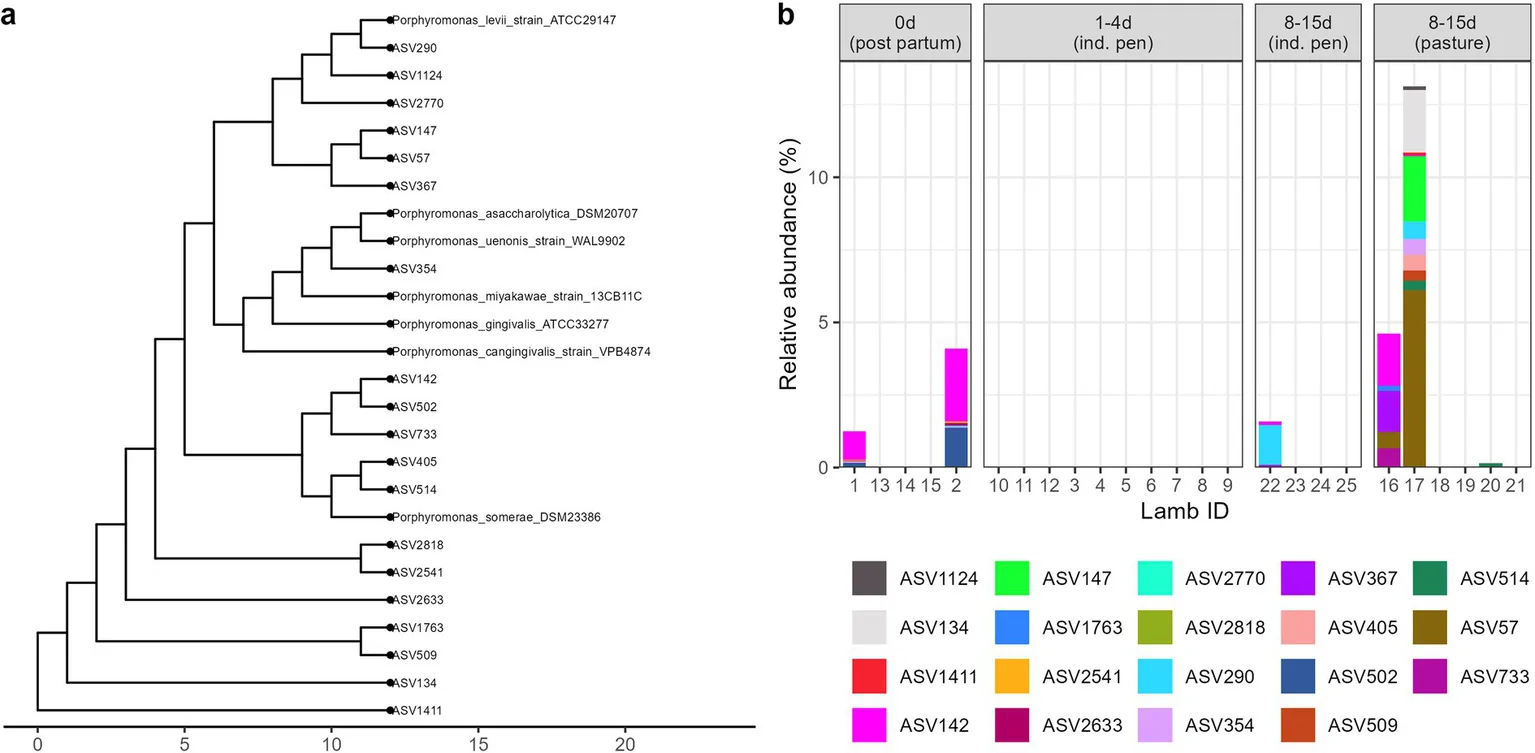
**(a)** Phylogenetic placement of *Porphyromonas* ASVs aligned with reference strains and **(b)** their abundances across age-location groups. ASV abundances are summarised by lamb and are shown relative to the total microbial community. Individual foot level abundances are shown in Supplementary Figure S2.

Forty-one ASVs were assigned to *Treponema*, but none showed similarity to pathogenic *Treponema* species previously associated with ovine foot disease (Supplementary Figure S3). Similarly, the four ASVs assigned as *Mycoplasma* shared ≤86% sequence identity with *Mycoplasma fermentans*, a taxon previously linked to footrot lesions (35), and were therefore considered unlikely to belong to this species.

## 4 Discussion

Here we present the first study and characterisation of the early-life IDSM of lambs within a commercial flock system. We show that neonatal lambs possess an IDSM that is characteristic of individuals, and that both bacterial load and microbiome complexity differ among age-location groups representative of those typically encountered during early life in commercial sheep production systems (Table 3). We also demonstrate that taxa associated with footrot are detectable in the IDSM during early life. We discuss the implications of these findings below.

The detection of *Porphyromonas* spp. in the early-life IDSM of lambs suggests that colonisation by taxa associated with footrot can occur shortly after birth. This finding indicates that exposure to these organisms may occur very early in life, potentially through maternal contact, the surrounding environment, or both. However, the data generated in the current study do not permit identification of the specific source of colonisation. *Porphyromonas* species have been found in the vaginal microbiome of ewes (36) supporting this as a possible source for seeding the newborn lamb IDSM. *Porphyromonas* ASVs were more diverse in the 8–15 day old lambs, suggesting further acquisition of these bacteria from the environment, although without longitudinal data it is not possible to detect persistence. The current study used 16S rRNA gene sequencing, which limits reliable taxonomic classification to the genus level. However, sequence alignment suggested that the *Porphyromonas* sequences in these lambs were most similar to *P. levii*, *P. somerae*, *P. ueononis* and *P. asaccharolytica*. Of these species only *P. asaccharolytica* has been linked to ovine footrot (14, 37), while *P. somerae* and *P. levii* have been associated with bovine footrot and bovine digital dermatitis (38, 39). Therefore, the ASVs detected in the current study are similar to *Porphyromonas* taxa that have been associated with ruminant foot diseases; however, their role in foot health and disease would require further investigation. Together, these findings indicate that colonisation by anaerobic taxa associated with footrot lesions may occur earlier than previously recognised. The detection of *Porphyromonas* spp. in the lamb IDSM is of particular interest given the hypothesis that presence of *Porphyromonas* spp. predisposes sheep to footrot (17). It would be of particular interest to determine whether potential pathogens seeded at birth persist in the IDSM and to quantify their relationship to future footrot risk for the lambs.

Unlike Muzafar et al. (6) we did not detect *D. nodosus* in any of the lambs sampled. We used qPCR to confirm this finding as there have been previously reported issues with detecting *D. nodosus* using 16S rRNA gene primers (40), although not for the V3-V4 primers used in this study. The absence of *D. nodosus* in this group of lambs was likely due to low prevalence of footrot within the ewes in this flock. Molecular studies indicate that *D. nodosus* is detected on feet with footrot lesions which then transiently contaminate the environment (21). Both low prevalence of footrot and dry lambing shed floors are known factors that reduce the prevalence of *D. nodosus* and therefore reduced likelihood of detection of *D. nodosus* on the lamb interdigital skin. In the study by Muzafar et al. (6), 39/40 ewes had detectable *D. nodosus* on their feet and *D. nodosus* was also prevalent in bedding, potentially suggesting high environmental contamination and subsequent likelihood of transfer to lambs; if ewes in our study had low, or no *D. nodosus* loads on their feet, lambs would have a much lower probability of having *D. nodosus* on their feet.

The observed variation in community composition across early life suggests that the lamb interdigital skin microbiota undergoes substantial changes during early colonisation, although longitudinal data would be required to confirm this suggestion. Within this study, associations with age and location could not be disentangled, but it is typical for lambs to move between several environments at a young age in commercial sheep farming. Environmental surfaces are likely to contribute to IDSM composition through both transient and persistent colonisation with bacteria from that environment. In addition, environmental conditions, for example moisture and pH, may influence individual species of bacteria present on feet. This may be through increased survival and transmission of certain bacteria, as has been shown for *D. nodosus* in wet conditions (21, 41). The potential association of IDSM composition with environment found in the current study agrees with previous findings showing an influence of season and weather on skin microbiota in adult sheep (17, 42). Together, these findings indicate that key components of the IDSM, including taxa previously associated with footrot, are established early in life. The observed associations with age and environment suggest that environmental exposures may contribute to microbial colonisation during this period; however, as potential source populations were not sampled, this cannot be confirmed from the current data. Nevertheless, these findings highlight early life as an important phase in IDSM development in sheep.

The initial close clustering of IDSM in neonatal lambs (Figure 2) suggests that initial seeding of the IDSM is relatively consistent across individuals. After this initial seeding, the skin is likely exposed to bacteria from a variety of sources, including different body sites of the ewe, siblings, other sheep in the flock, and the environment, which may contribute to the greater variability in IDSM composition observed in older lambs. The relative influence of these sources, and the extent to which bacteria from each persist over time, require further investigation. This pattern of relatively low variability in neonatal lambs and greater heterogeneity in older lambs indicates that early life represents a key period in IDSM establishment, during which microbial communities are still being structured. In humans, maternal skin microbiota can have persistent effects on the infant skin microbiota even at one year of age (43), demonstrating that early microbial exposures may have long-lasting influence on microbiome composition.

One possible mechanism underlying the observed variability in IDSM between newborn and older lambs within this study is social transmission of microbes between animals. The ‘social microbiome’ theory (44) proposes that some microbes are transmissible through social contact, meaning microbial communities reflect host social connections. If bacteria are shed into the environment from the dam’s feet, lambs that spend more time with their mother, e.g., single lambs compared with twin lambs (18), may be more likely to acquire a microbiota resembling that of the ewe. This may contribute to the heterogeneous effects of age / location observed in this study. Lambs tend to make less contact with their mothers as they age (45, 46), potentially increasing the influence of other social and environmental sources. Longitudinal studies would be valuable to determine whether a core IDSM persists over time despite changing environments and social interactions. At the single time-point examined here, approximately one third of the variation in α-diversity was attributable to differences between lambs; this may change over time as peer-peer contact increases, as seen in human infants (47).

*Corynebacteriaceae, Moraxellaceae, Actinomycetaceae* and *Staphylococcaceae* have been reported as the dominant bacterial families in the IDSM of healthy adult sheep (15, 16, 37, 48). Our results show a similar pattern for lambs, with *Corynebacteriaceae* and *Staphylococcaceae* prevalent across all age groups and locations. Lambs sampled in this study had a lower number of different microbial taxa present (indicated by IDSM richness) than has been reported for reported for adult sheep in other studies (16, 48), however the overall balance and variety of these microbes (as measured by Shannon index) was comparable to that reported for the healthy IDSM of adult sheep (14, 16). In humans, alpha diversity of infant skin microbiota is known to be lower than that of adults (49), whereas this is not the case for many wildlife species including bank voles (50), zebra finches (51) and fur seals (52, 53).

Whilst this study provides novel insights into the early-life IDSM in lambs, it is from a small group of lambs on one farm. Farm is known to impact the composition of the IDSM in adult sheep (54) therefore differences in IDSM composition of lambs would be expected across farms, especially given the known role of environment in determining skin microbiota in sheep (17, 42). Despite this, there is consistency across farms and countries in broader patterns, such as the core families making up the healthy IDSM (15, 16, 55). Further work is needed to understand the extent to which the identified core IDSM for lambs persists into later life given that microbial communities can change over time and are influenced by social connections other than maternal connections (46, 47, 56). Longitudinal studies linking early-life IDSM composition to subsequent footrot outcomes will be essential to determine whether early microbial exposure contributes to lifetime disease risk.

## 5 Conclusion

In this study we have provided the first evidence for and description of the early-life IDSM of lambs. We have shown that bacterial load is higher in lambs ≥1 day old in individual pens and at pasture compared with 0-day old lambs and composition of the IDSM varies between lamb age-location groups. We have also demonstrated that bacterial pathogens implicated in footrot pathogenesis are detected in the IDSM of some lambs, demonstrating the need to determine whether potential pathogens seeded around birth persist in the IDSM and influence future risk of footrot in those feet.

## Data Availability

Sequencing data from this study are available from the NCBI Sequence Read Archive (BioProject PRJNA1435082), https://www.ncbi.nlm.nih.gov/bioproject/PRJNA1435082.
