# Supplementary material for: Variation in the early-life interdigital skin microbiota of lambs in a commercial sheep flock
Source: Front Vet Sci. 2026 Sep 30;13:1923848. doi: 10.3389/fvets.2026.1923848 (PMC13662760; doi:10.3389/fvets.2026.1923848)
Supplement: Supplementary file 1 [file Supplementary_file_1.docx]

Supplementary Material: Variation in the early-life interdigital skin microbiota of lambs in a commercial sheep flock

**Supplementary Tables**

**Table S1 Details of lambs included in the study including ewe identity, lamb age, sex and location**

| **Lamb number** | **Ewe number** | **Lamb age (days)** | **Lamb sex** | **Location** | **Comments** |
| --- | --- | --- | --- | --- | --- |
| 1 | 1 | 0 | M | NA (post partum) |  |
| 2 | 1 | 0 | M | NA (post partum) |  |
| 3 | 2 | 3 | M | Individual pen |  |
| 4 | 3 | 2 | F | Individual pen |  |
| 5 | 4 | 4 | F | Individual pen |  |
| 6 | 4 | 4 | F | Individual pen |  |
| 7 | 15 | 2 | M | Individual pen |  |
| 8 | 16 | 3 | F | Individual pen |  |
| 9 | 17 | 1 | M | Individual pen |  |
| 10 | 18 | 1 | M | Individual pen |  |
| 11 | 19 | 3 | M | Individual pen |  |
| 12 | 20 | 2 | F | Individual pen |  |
| 13 | 5 | 0 | M | NA (post partum) |  |
| 14 | 5 | 0 | M | NA (post partum) |  |
| 15 | 6 | 0 | M | NA (post partum) |  |
| 16 | 7 | 12 | M | Pasture |  |
| 17 | 8 | 15 | F | Pasture |  |
| 18 | 9 | 10 | F | Pasture |  |
| 19 | 10 | 12 | F | Pasture |  |
| 20 | 11 | 14 | F | Pasture |  |
| 21 | 12 | 14 | M | Pasture |  |
| 22 | 13 | 8 | F | Individual pen | Had been at pasture |
| 23 | 13 | 8 | F | Individual pen | Had been at pasture |
| 24 | 14 | 9 | F | Individual pen | Had been at pasture |
| 25 | 14 | 9 | M | Individual pen | Had been at pasture |

- M = male, F = female, NA = not applicable (lambs had not yet touched the ground at the point of sampling)

**Table S2 Primers used in the study**

| Name | Target | Sequence | Reference |
| --- | --- | --- | --- |
| SD-17F | 16S rRNA V3/V4 | CCT ACG GGN GGC WGC AG | (Klindworth et al., 2013) |
| SD-21R |  | GAC TAC HVG GGT ATC TAA TCC |  |
| rpoD F | *D. nodosus* *rpoD* | GCTCCCATTTCGCGCATAT | (Witcomb et al., 2014) |
| rpoD R |  | CTGATGCAGAAGTCGGTAGAACA |  |
| rpoD probe |  | 6FAM-TCGAACATCTCTCGCTTTTTCCCCGA-BBQ |  |

**Supplementary figures**

**Figure S1 Rarefaction curves showing observed species richness (number of ASVs) by sequencing depth (number of reads).** (a) rarefaction curves for 96 samples with > 1000 reads. (b) two samples with high read counts (>100,000 reads) have been removed so that remaining samples can be visualised more clearly. For these 94 samples, median reads per sample was 15,834 (IQR 10,036 - 23,913).

**Figure S2** **Abundance of *Porphyromonas* ASVs across age-location groups.** ASV abundances are shown at foot level and relative to the total microbial community. Feet are grouped by lamb with lamb ID shown in the lower of the two panels above the plot. Lamb age-location group is shown in the upper panel above the plot.

**Figure S3 Phylogenetic placement of *Treponema* ASVs aligned with reference strains from Genbank.** Pathogenic strains previously implicated in ovine foot disease (*T. pedis, T. phagedensis, T. medium, T. denticola and T. putidum*) form a distinct cluster. The ASVs from this study cluster show greatest similarity to *T. brennaborense*, *T. bryantii,* *T. berlinense* and *T. porcinum*.

**Supplementary methods**

1. **DNA extraction from swabs**

DNA was extracted from swabs using the ZymoBIOMICS DNA/RNA Miniprep kit (Zymo Research) as follows. Swabs were defrosted and transferred to 750ul of DNA Shield solution in bead beating tubes using sterile tweezers. Bead beating was performed for 2.5 minutes at a frequency of 30^-s^, followed by a 1-minute pause, then a further 2 minutes of bead beating. Swabs were centrifuged at 13,000 x g for 30 seconds and then 400 μl of supernatant was combined with 800 μl lysis buffer and loaded onto DNA columns in two 600 μl aliquots. Prepping and washing of the DNA column was performed according to the manufacturer's instructions and DNA was eluted in 50 μl nuclease free H_2_0.

2. **Quantitative PCR protocols**

Quantification of total bacteria: All reactions were carried out in a final volume of 10 μl using 2 × Sensifast SYBR Lo-ROX Mastermix (Meridian Bioscience, Scientific Laboratory Supplies, Nottingham, UK). Cycling conditions consisted of 1 cycle of 95°C for 3 minutes followed by 40 cycles of 95°C for 5 seconds and 62°C for 20 seconds.

Quantification of *Dichelobacter nodosus:* All reactions were carried out in a final volume of 25 μl using 2 × TaqMan^®^ Universal Mastermix (Applied Biosystems, Thermofisher Scientific, Warrington, UK). Cycling conditions consisted of 1 cycle at 50°C for 2 minutes, 1 cycle at 95°C for 10 minutes followed by 40 cycles at 95°C for 15 seconds and 55°C for 1 minute.

All quantitative PCRs were performed in 96 well plates (Applied Biosystems, Thermofisher Scientific, Warrington, UK) with standard curve samples and a negative control (nuclease free H_2_0) included in each plate in triplicate. Samples were quantified in duplicate using the standard curve and then a mean value taken. Samples were classified as positive when both replicates were positive.

3. **Library prep for 16S rRNA gene sequencing**

All samples and controls underwent amplicon PCR clean-up using 0.8X AMpure beads (Beckman; A63882). Illumina dual index barcodes were added to each library using an Index PCR (Illumina XT Index Kit v2, Set C: FC-131-2003) followed by PCR clean up with 1X AMpure beads (Beckman; A63882). All samples underwent 8 cycles of PCR.

Libraries were quantified using the Qubit Fluorometer and the Qubit dsDNA HS Kit (Thermo-Fisher Scientific; Q32854). Library fragment-length distributions were analyzed using the Agilent TapeStation 4200 and the Agilent HS D1000 ScreenTape Assay (Agilent; 5067-5584 and 5067-5585). Libraries were then pooled in equimolar amounts. Final library pool quantification was performed using the KAPA Library Quantification Kit for Illumina (Roche; KK4824).

**References**

KLINDWORTH, A., PRUESSE, E., SCHWEER, T., PEPLIES, J., QUAST, C., HORN, M. & GLÖCKNER, F. O. 2013. Evaluation of general 16S ribosomal RNA gene PCR primers for classical and next-generation sequencing-based diversity studies. *Nucleic Acids Res,* 41**,** e1.

WITCOMB, L. A., GREEN, L. E., KALER, J., UL-HASSAN, A., CALVO-BADO, L. A., MEDLEY, G. F., GROGONO-THOMAS, R. & WELLINGTON, E. M. H. 2014. A longitudinal study of the role of *Dichelobacter nodosus* and *Fusobacterium necrophorum* load in initiation and severity of footrot in sheep. *Preventive Veterinary Medicine,* 115**,** 48-55.
